# Wilder than intense: higher frequency, variability, and viral flows of porcine circovirus 3 in wild boars and rural farms compared to intensive ones in northern Italy

**DOI:** 10.3389/fmicb.2023.1234393

**Published:** 2023-07-31

**Authors:** Giovanni Franzo, Giulia Faustini, Matteo Legnardi, Giacomo Berto, Mariangela Dal Maso, Viviana Genna, Maria Luisa Menandro, Francesca Poletto, Mattia Cecchinato, Michele Drigo, Claudia Maria Tucciarone

**Affiliations:** ^1^Department of Animal Medicine, Production and Health, University of Padova, Legnaro, Padua, Italy; ^2^AULSS 8 Berica, Dip di Prevenzione, Servizi Veterinari, Vicenza, Italy; ^3^Azienda Ulss 9 Scaligera, Verona, Italy

**Keywords:** PCV-3, wild boar, rural pig, molecular epidemiology, phylogenetics, virus

## Abstract

**Introduction:**

Porcine circovirus 3 (PCV-3) was firstly reported in 2017. Although evidence of its pathogenic role has been provided, its clinical relevance seems lower than Porcine circovirus 2 (PCV-2), as well as its evolutionary rate. Different studies have reported a high PCV-3 prevalence in wild boars, sometimes higher than the one observed in commercial pigs. Nevertheless, to date, few studies have objectively investigated the relationships between these populations when inhabiting the same area. Moreover, the role of small-scale, backyard pig production in PCV-3 epidemiology is still obscure.

**Methods:**

The present study investigated PCV-3 occurrence in 216 samples collected from the same area of Northern Italy from commercial and rural pigs, and wild boars. PCV-3 presence was tested by qPCR and complete genome or ORF2 sequences were obtained when possible and analysed using a combination of statistical, phylogenetic and phylodynamic approaches.

**Results:**

A higher infection risk in wild boars and rural pigs compared to the commercial ones was demonstrated. The phylodynamic analysis confirmed a larger viral population size in wild and rural populations and estimated a preferential viral flow from these populations to commercial pigs. A significant flow from wild to rural animals was also proven. The analysis of the Italian sequences and the comparison with a broader international reference dataset highlighted the circulation of a highly divergent clade in Italian rural pigs and wild boars only.

**Discussion:**

Overall, the present study results demonstrate the role of non-commercial pig populations in PCV-3 maintenance, epidemiology and evolution, which could represent a threat to intensive farming.

## 1. Introduction

The number and economic relevance of rural-backyard pig farming have gradually declined over decades in Italy due to the progressive pig farming industrialization (Maiorano, 2009; Augere-Granier, 2020). Most of the pig production is organized according to a strong hierarchical structure, with several productive pig flows. However, some rural farms still persist, both for self-consumption and small-scale production. In recent years, the increasing consumers’ request for natural, organic, antibiotic free products has given new life to this sector, opening new market opportunities (Di Pasquale et al., 2014; Clonan et al., 2015). Because of the small production, the managerial level and resources for high-level biosecurity and infection control measure application are often limited. Therefore, backyard farms could represent a threat to the industrial sector, harboring, maintaining and potentially affecting the evolution of several pathogens that can then spread to the industrial system, as previously modeled for porcine reproductive and respiratory syndrome virus (PRRSV) and other infections of veterinary interest (Franzo et al., 2020b, 2021a). The different features of animal farming might also create peculiar ecological niches favoring certain pathogens or variants instead of others (Franzo et al., 2015; Molini et al., 2021). Finally, because of the facility structure, limited external biosecurity, and often geolocalization near forests or uncultivated areas, contacts with wild animals are more likely, and pathogen exchange can occur (Jara et al., 2021).

Porcine circovirus 3 (PCV-3) has emerged as a new likely pathogen of swine (Palinski et al., 2017). PCV-3 is a non-enveloped virus, a member of the family *Cirvoviridae*, genus *Circovirus*. It features a circular genome of approximately 2Kb containing two main open reading frames (ORFs): ORF1 encodes the Rep protein, participating in the viral genome replication, and ORF2 codes for the Cap one, the only constituent of the viral capsid (Klaumann et al., 2018; Opriessnig et al., 2020). Because of the higher variability, ORF2 has been the main target for molecular epidemiological studies and strain classification, although it must be stressed that PCV-3 variability and evolutionary rate are significantly lower than Porcine circovirus 2 (PCV-2) ones (Franzo et al., 2019b, 2020a).

Initially discovered in the United States (2015) by next-generation sequencing (NGS) methods in swine affected by respiratory and neurological signs, cardiac and multisystemic inflammation, reproductive failure and porcine dermatitis and nephropathy syndrome (PDNS)-like conditions (Phan et al., 2016; Palinski et al., 2017), it was thereafter reported worldwide. Since then, the virus has been detected in pigs in presence of several clinical–pathological outcomes, such as respiratory disease, digestive disorders, congenital tremors, rectal prolapse, reproductive problems and multisystemic inflammation (Klaumann et al., 2018). However, this novel virus has been detected in healthy animals of different ages and countries as well (Stadejek et al., 2017; Zhai et al., 2017; Klaumann et al., 2018, 2019; Franzo et al., 2018c). Therefore, its pathogenic role, although rapidly accepted by the veterinary community, was long uncertain. Increasing evidence has nevertheless led to the case definitions for PCV-3-reproductive disease and PCV-3-systemic disease at least (Saporiti et al., 2021). Similarly to PCV-2, the infection prevalence, although variable, is often high in commercially raised pigs and wild boars as well, in which the infection frequency can be as high or even higher than in the domestic ones. A certain strain exchange, with unknown dominant directionality, between the two populations has also been demonstrated (Franzo et al., 2018d, 2019a; Prinz et al., 2019; Czyżewska-Dors et al., 2020).

Currently, there are scarce data on the frequency and features of PCV-3 strains in the rural pig population. Therefore, the epidemiological role of this population and how it might interact with the above-mentioned ones are unknown. In the present study, several samples originating from intensively raised pigs, rural ones, and wild boars from Northern Italy were tested for PCV-3 detection and molecular characterization was attempted on positive samples to depict the occurrence, frequency of infection and interface among these populations.

## 2. Materials and methods

### 2.1. Sampling

To allow a comparison among strains circulating in the same area and evaluate potential local transmission networks, samples were collected from the neighboring Lombardy and Veneto regions of Northern Italy.

More in detail, rural samples originated from pigs reared in small family backyard farms (hereafter referred to as rural farms) belonging to different municipalities and regularly slaughtered for self-consumption or sales at local markets between 2021 and 2022. Sampled animals were visited ante-mortem, and post-mortem inspection was conducted by official veterinarians, according to the European legislation, and lung and lymph node samples were collected. Farm location and sampling date were recorded (Supplementary Table S1).

Intensively raised pig samples were selected among archive samples routinely collected from the same area by private companies for monitoring activities or from pigs showing clinical signs and whose PCV-3 status was unknown.

Wild boar samples (lung samples and lymph nodes) were obtained from subjects hunted or killed during the routine culling campaign performed in 2022 in Verona and Vicenza (Veneto) provinces. Tissues were collected by official veterinarians and anamnestic data were provided by hunters by filling out a dedicated, mandatory form.

All biological samples were stored at −80°C until DNA extraction.

### 2.2. PCV-3 extraction and detection

Lungs and lymph nodes from pigs and wild boars were processed mechanically for homogenization after adding 10 mL of PBS 1X (phosphate buffer saline) per gram of tissue.

DNA was extracted from 100 μL of sample homogenate using the Viral DNA/RNA kit (A&A Biotechnology, Gdańsk, Poland) according to the manufacturer’s instructions. Extracted DNA was stored at-80°C until further processing. PCV-3 genome presence was tested and quantified using the qPCR assays described in Franzo et al. (2018a).

ORF2 amplification was attempted on positive samples using the primer pairs PCV3_1303F (5-ACCGGAGGGGTCAGATTTAT-3) and PCV3_8R (5-TGCCGGGTAATACTAGCC3-3) (Franzo et al., 2019a) while the primer pairs reported in Franzo et al. (2018b) were used for complete genome characterization. Biometra TAdvanced® Thermal Cycler (Analytik Jena GmbH, Jena, Germany) and Invitrogen™ Platinum™ II Taq Hot-Start DNA polymerase (Thermo Fisher Scientific, Waltham, MA, USA) were used to perform all the PCRs. Two μL of DNA were added to a reaction mix including 1X Platinum™ II PCR buffer, 0.6 μM of each primer, 0.2 mM of each dNTP and one unit of Platinum™ Taq Hot-Start DNA Polymerase. Molecular grade water was added up to a total volume of 25 μL. The thermal protocol involved an activation phase at 95°C for 2 min, followed by 45 cycles at 94°C for 15 s, 60°C for 15 s and 68°C for 15 s. A 1 min final elongation was also included. Amplification and band specificity were checked by SYBR-safe stained agarose gel electrophoresis, and all positive amplicons were purified using Applied Biosystems® ExoSAP-IT PCR Product Cleanup Reagent (Thermo Fisher Scientific, Waltham, MA, USA). The same PCR primers were used to Sanger-sequence the amplicons in both directions at Macrogen Europe (Milan, Italy).

### 2.3. Sequence analysis

Chromatogram quality was evaluated by FinchTV (2004–2006 Geospiza Inc) and consensus sequences assembly was performed with ChromasPro (ChromasPro Version 2.0.0, Technelysium Pty Ltd., South Brisbane, Australia). A collection of complete ORF2 and full genomes of PCV-3 strains were downloaded from GenBank. Metadata on collection country, host and date were annotated on the sequence name when available. Unverified sequences and those of poor quality, including premature stop codons, frameshift mutations, unknown bases or obvious misalignments were removed from the dataset. Partial capsid sequences obtained from Italian wild boars were also downloaded for comparison purposes. Moreover, animal category and sampling location were added to previously submitted Italian sequences when available.

All the obtained complete ORF2 sequences were aligned at the protein level using MUSCLE (Edgar, 2004) method implemented in MEGA X (Kumar et al., 2018) and then backtranslated as nucleotides. Complete genome sequences were aligned using MAFFT (Standley, 2013). Recombination occurrence was tested on both datasets using the Genetic Algorithm for Recombination Detection method (GARD) implemented in Datamonkey (Kosakovsky Pond et al., 2006; Weaver et al., 2018).

Maximum likelihood (ML) phylogenetic trees were reconstructed using IQTree (Nguyen et al., 2015), selecting the substitution model with the lowest Bayesian Information Criterion (BIC).

### 2.4. Structured coalescent analysis

The Bayesian structured coalescent analysis implemented in the package MultitypeTree of BEAST 2.7 (Vaughan et al., 2014; De Maio et al., 2015; Bouckaert et al., 2019) was used to jointly estimate the viral effective population size (*Ne*) and migration rates among animal categories (i.e., commercial, rural and wild animals), together with other population parameters and phylogenetic trees representing viral genealogy. Briefly, the structured coalescent is a statistical model that describes the genealogy of individuals sampled from a structured population that evolves according to a migration matrix model. Different categories can thus be seen as islands (demes) of different population size, connected by migration events that occur at a constant rate over time. A 100 million generation Markov chain Monte Carlo (MCMC) run was performed on the dataset including all Italian ORF2 sequences originating from subjects whose category was known, sampling model parameters and trees every 10,000 generations. The nucleotide substitution model was selected based on Bayesian Information Criterion (BIC), while a relaxed lognormal molecular clock was implemented as previously described (Franzo et al., 2019b; Cui et al., 2022). Results were visually inspected using Tracer 1.5 (Rambaut and Drummond, 2013) and accepted only if mixing and convergence were adequate and the Estimated Sample Size was greater than 200 for all parameters. Parameter estimation was summarized in terms of mean and 95% Highest Posterior Density (HPD) after the exclusion of a burn-in equal to 20% of the run length. Maximum clade credibility (MCC) trees were constructed and annotated using Treeannotator (BEAST package).

### 2.5. Selection analysis

A contrast-FEL (Kosakovsky Pond et al., 2021) analysis was performed on Italian Cap sequences to test the presence of differential selective pressures acting on rural and commercial pigs compared to wild boars, assumed as a reference.

### 2.6. Statistical analysis

The odds ratio of infection and relative confidence intervals were calculated fitting a logistic regression model having the PCV-3 qPCR results as the dependent variable and the animal category (commercial (baseline), rural or wild boar) as an independent one. Differences in viral titres of positive samples were evaluated among animal categories by ANOVA method. Titres were log10 transformed to respect the normality and homoscedasticity assumptions. The statistical significance level was set at *p* < 0.05. All analyses were performed using R 4.2.2.

## 3. Results

### 3.1. Samples and PCV-3 qPCR results

A total of 216 samples were included in the study, 68 and 45 rural and commercial pigs, and 103 wild boars. Of those, 47 (69.12% [95CI: 56.74–79.76%]), 11 (24.44% [95CI: 12.88–39.54%]) and 58 (56.31% [95CI: 46.18–66.06%]) tested positive, respectively. The odds of being PCV-3 positive were (6.91 [95CI: 3.03–16.82]; *p* < 0.001) and 3.98 [95CI: 1.86–9.04] higher in rural pigs and wild boars compared to commercial pigs.

Although the mean titres (genome copy number) were lower in commercial pigs, the difference among pig categories was not statistically significant (*p* = 0.5925).

### 3.2. Sequence analysis

Nineteen complete genomes and 22 ORF2 sequences were obtained (acc. numbers OQ754379-OQ754419). The mean genetic distance (p-distance) among Italian strains (including those obtained in the present study and previously available) was 0.014 [interval: 0–0.092] and 0.012 [interval: 0.001–0.046] at ORF2 and complete genome level, 0.05 [interval: 0–0.12] and 0.006 [interval: 0.001–0.008] among commercial pigs, 0.014 [interval: 0.000–0.039] and 0.014 [interval: 0.001–0.034] among rural pigs and 0.016 [interval:0.000–0.092] and 0.016 [interval: 0.000–0.092] among wild boars.

At Cap amino acid level, the distance (*p*-distance) among the Italian strain was 0.014 [interval: 0–0.131], 0.004 [interval: 0.000–0.014] among commercial pigs, 0.011 [interval: 0.000–0.033] among rural pigs and 0.017 [interval: 0.000–0.131] among wild boars (Table 1).

**Table 1:** Summary of genetic and amino acid distances calculated between strain pairs sampled from different animal categories.

Target	Category	Region	Mean	Interval
DNA	Overall	ORF2	0.014	0–0.092
		Complete genome	0.012	0.001–0.046
	Commercial	ORF2	0.05	0–0.12
		Complete genome	0.006	0.001–0.008
	Rural	ORF2	0.014	0.000–0.039
		Complete genome	0.014	0.001–0.034
	Wild boars	ORF2	0.016	0.000–0.092
		Complete genome	0.016	0.000–0.092
Amino Acid	Overall	ORF2	0.014	0–0.131
	Commercial	ORF2	0.004	0.000–0.014
	Rural	ORF2	0.011	0.000–0.033
	Wild boars	ORF2	0.017	0.000–0.131

No evidence of differential selective pressures acting among the considered categories was detected using contrast-FEL.

### 3.3. Phylogenetic analysis

**Figure 1:**
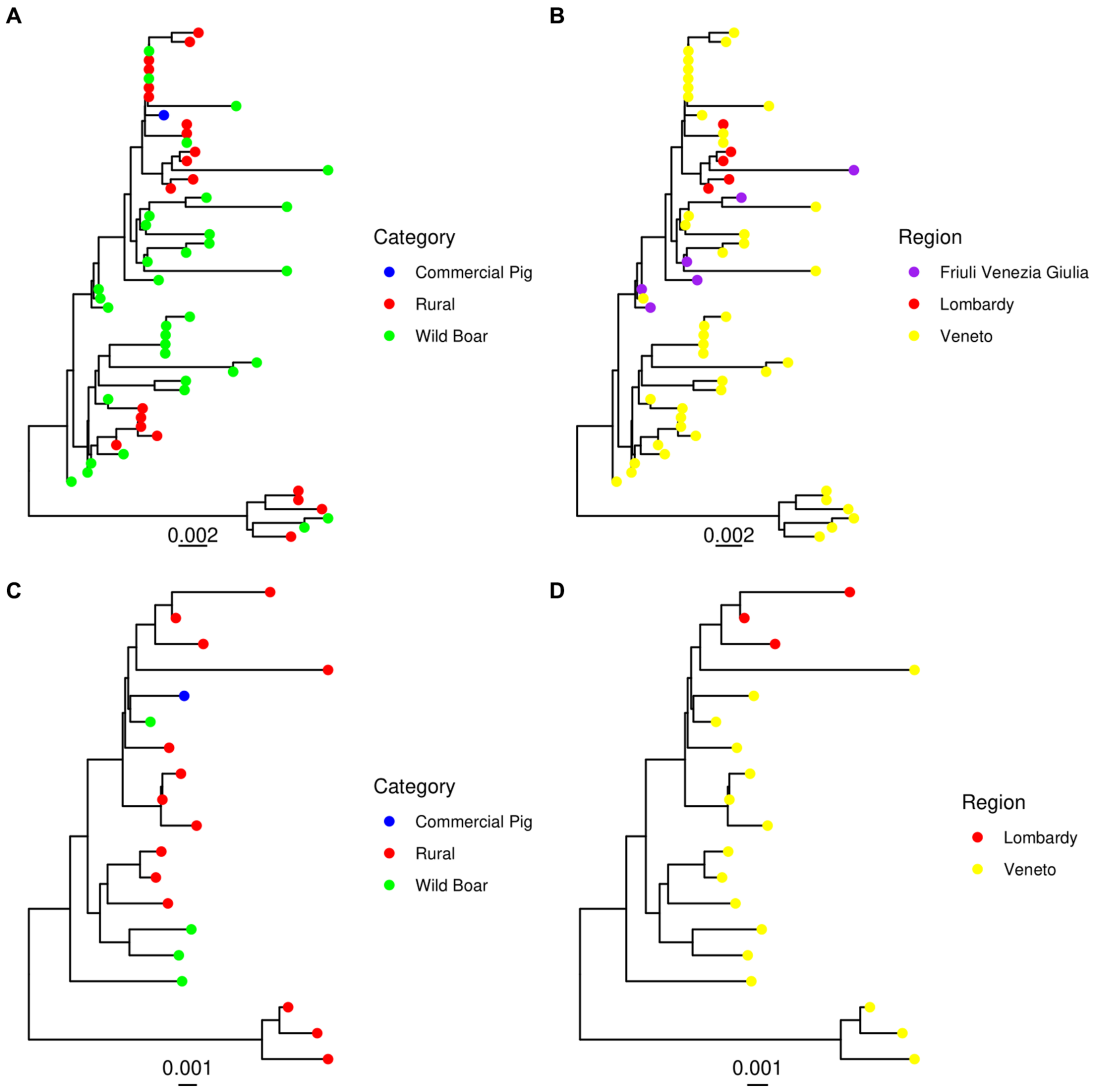
Maximum likelihood phylogenetic tree reconstructed based on the ORF2 **(A,B)** and complete genome **(C,D)** of the strains sequenced in the present study. The animal category **(A,C)** and geographical location **(B,D)** have been color-coded.

**Figure 2:**
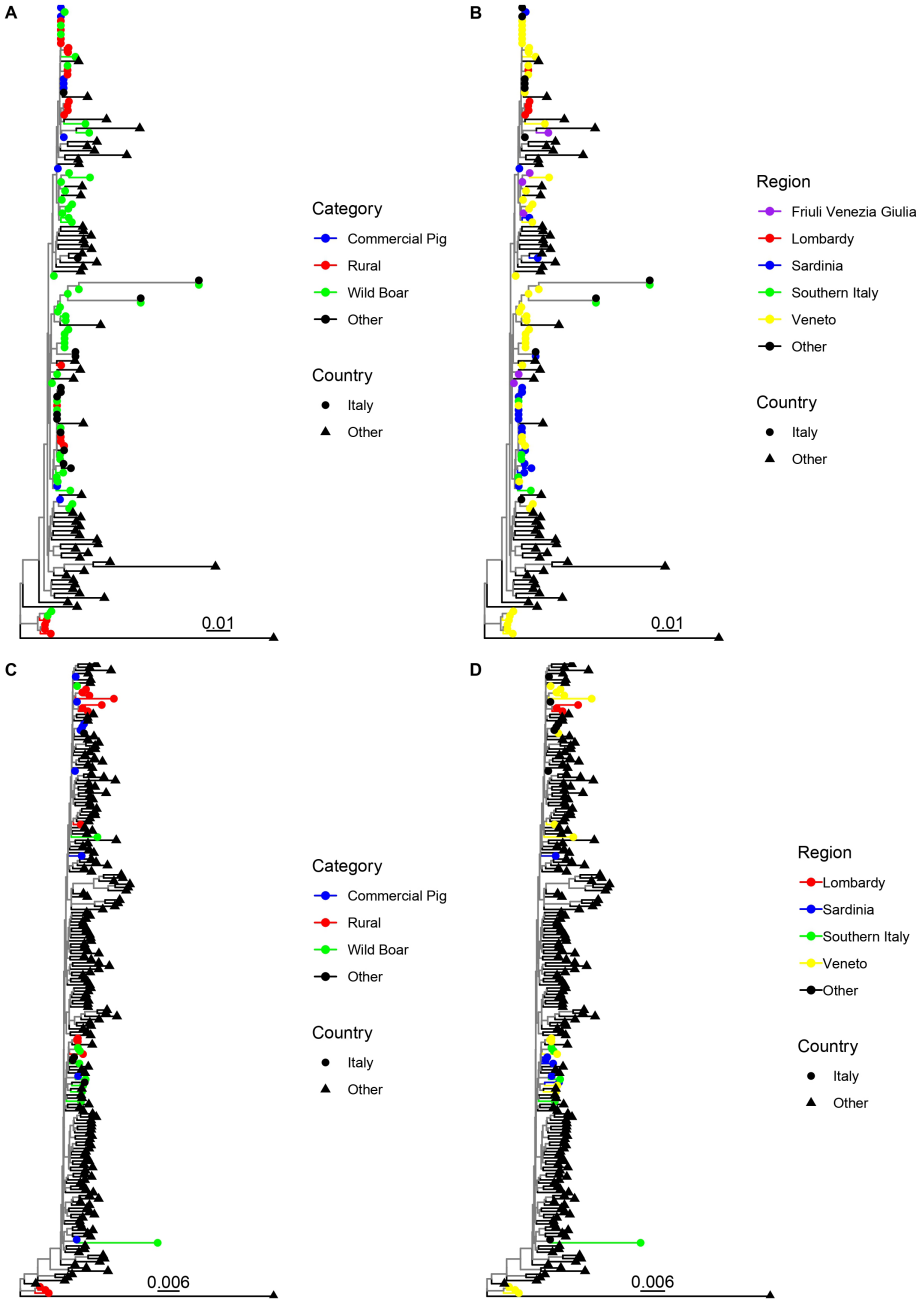
Maximum likelihood phylogenetic tree reconstructed based on the ORF2 **(A,B)** and complete genome **(C,D)** of the strains sequenced in the present study plus a set of reference sequences. The animal category **(A,C)** and geographical location **(B,D)** have been color-coded. A more detailed description (including strain names annotated with collection host, country and date) of the relationship between the considered strains is provided in Supplementary Figure S1.

No evidence of recombination among the considered Italian strains was detected by GARD. The phylogenetic analysis of the sequences obtained by our group in Northern Italy revealed the presence of two main clades using both the ORF2 and complete genome dataset. The main clade included sequences from all the sampled regions and animal categories, while the second, more divergent one, comprised strains from wild boars and rural pigs only, sampled in Veneto region. For this clade, the complete genome was only obtained from strains derived from rural animals (Figure 1). Comparable results were obtained when all Italian sequences were analyzed together with international reference sequences (Figure 2). The minor clade was different from other PCV-3a strains previously described, although it did not fulfil the criteria to be defined as a new genotype since the minimum genetic distance between these strains and members of the PCV-3a genotype was 0.016 (strain MZ667335.1|Susscrofa|China|2021-03-11) at the complete genome level and 0.026 (strains MK044780.1|WildBoar|FriuliVeneziaGiulia|Italy|2017) at ORF2 level. A Chinese strain (ON184639.1|Susscrofadomesticus|China|2021) was also part of this clade (*p* = 0.009 compared to other Italian ones). Overall, although a certain tendency of Italian strains to cluster together, different clades were interspersed in the phylogenetic tree and foreign strains were part of predominantly Italian clusters (Figure 2). Within Italy the geographical clustering, although present, was even less apparent. In fact, different strains could be located in the same region, while identical strains were sampled from different areas. A tendency to group according to the animal category was observed, although also in this case clusters of closely related strains sampled from wild and rural subjects, wild and commercial and a combination of the three categories were present (Figure 1).

### 3.4. Structured coalescent analysis

**Figure 3:**
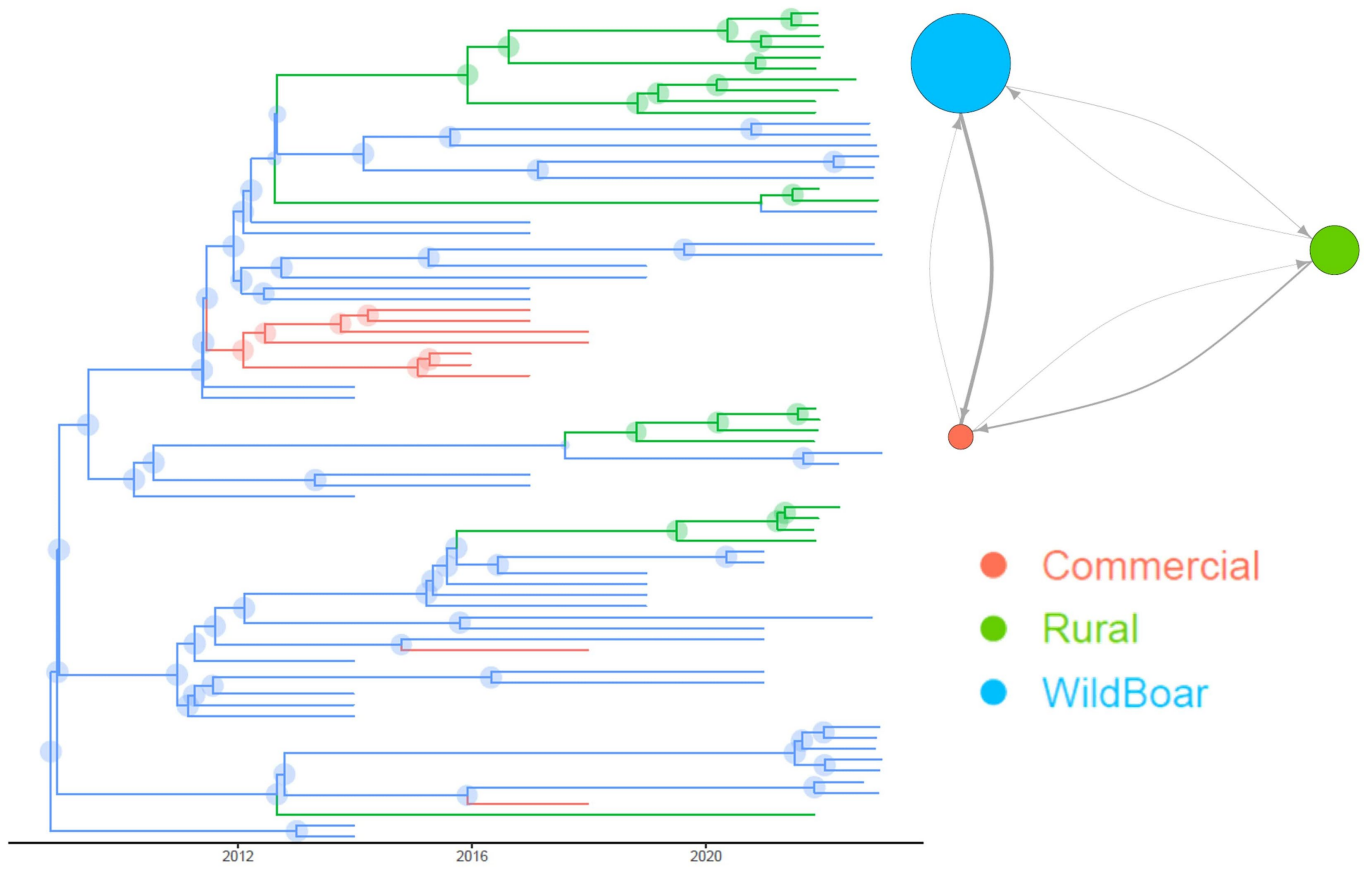
Structured coalescent-based phylogenetic tree of the Italian samples included in the present study. Branch colors, as from legend, mark the inferred animal category where the ancestral strain was circulating, while node size represents the posterior confidence of the inference. In the top right insert it is reported the network depicting the migration rate between animal categories. Arrows and circles size are proportional to the inferred migration rate and population size, respectively.

The evaluation of the population parameters, estimated through structured coalescent analysis revealed a high mutation rate (i.e. 1.49∙10^−3^; 95HPD: 5.33∙10^−4^–6.44∙10^−3^). The time to Most Recent Common Ancestor (tMRCA) of considered Italian strains was estimated in 1973 (95HPD 1964.20–1985.45). The structured coalescent analysis estimated relevant strain migration rates between groups. Migration rates from wild boars to commercial pigs and rural to commercial pigs were approximately 8 and 4 times higher than from commercial to rural pigs, respectively. Finally, strain migration from wild boars toward rural animals was twice as high (Figure 3).

The estimated population sizes of PCV-3 in wild boars and rural pigs were about 4 and 2 times bigger than that circulating in commercial swine.

## 4. Discussion

The recent identification of PCV-3 has raised great concern among researchers and veterinarians, likely because of the previous experience with PCV-2 and the association of at least two syndromes with PCV-3 infection (Segalés et al., 2013; Franzo et al., 2019b; Saporiti et al., 2021). Moreover, the genetic diversity of this species, as well as its evolutionary rate, have been estimated lower than those of PCV-2 (Franzo et al., 2019b, 2020a). Because of the similarities between the two viruses, such disparity is hard explain. The genetic diversity of a pathogen can be determined by a complex interaction among mutation rate, size of the viral population and selective pressure strength acting on it. A potential hypothesis could thus be the preferential presence of PCV-3 in niches, like wild and rural animals, allowing for a relatively smaller viral population size or posing less intense selective pressures. Recently, the role of rural pigs in the PRRSV and PCV-2 epidemiology has been investigated in Italy (Franzo et al., 2021a; Faustini et al., 2023), suggesting a relevant part in the maintenance and spreading of the infection. For PCV-2, minor genotypes have often been reported in unexpected host populations (e.g., rural and feral pigs, and wild animals) and/or geographical areas where industrial farming is marginal or in its infancy (Franzo et al., 2015, 2021b; Molini et al., 2021). It has tentatively been suggested that such ecological niches might favor the maintenance of less virulent genotypes, less fit to compete with the major ones that are more adapted to the commercial pig population. In fact, PCV-2c and-2e have been described at high frequency in these populations (Franzo et al., 2015; Faustini et al., 2023).

The PCV-3 scenario identified in the present study based on the Italian situation mirrors and confirms what was previously proposed (Franzo et al., 2015, 2021b; Molini et al., 2021; Faustini et al., 2023). Significantly higher odds of infection were demonstrated among wild and rural swine compared to commercial ones living in the same geographic area. While higher biosecurity measures could be involved, a comparable scenario was not observed for PCV-2 (Faustini et al., 2023), lessening their potential impact. The PCV-3 frequency in commercial pigs herein reported is in line with what reported by previous literature, thus a lack of diagnostic sensitivity in the considered context appears unlikely (Klaumann et al., 2018). Therefore, the preferential circulation of PCV-3 in non-commercial pigs might be speculated. Previous studies reported a high or even higher PCV-3 positive rate in wild boars compared to domestic animals (Franzo et al., 2018d, 2019a; Prinz et al., 2019; Czyżewska-Dors et al., 2020). Remarkably, these results were confirmed by structured coalescent analysis also, where bigger *Ne* was estimated for these populations based on genetic data analysis only. The causes behind this evidence are elusive. Similarly, it is currently unknown if this represents a peculiarity of the Italian scenario or if it can be generalized, and further investigations will be necessary.

Nevertheless, based on these observations, the lower evolutionary rate of PCV-3 compared to PCV-2 might be ascribable to its circulation in populations with lower density, less intense contacts, turnover and stressors, and rarer coinfection with multiple strains [which could facilitate recombination events and prompt evolution according to the red queen hypothesis (Clarke et al., 1994)], leading to stronger evolutionary bottlenecks, smaller population size and selective pressures.

The epidemiological relevance of rural/wild populations cannot be neglected for several reasons. Firstly, their contribution to the maintenance and spreading of the disease has been reported and the results of the present study are in line with and further strengthen the previous pieces of evidence (Franzo et al., 2021a; Faustini et al., 2023). Sequences collected from different pig categories were often part of the same cluster. Moreover, the structured coalescent analysis estimated a higher migration flow from wild and rural to commercial animals. A high viral exchange from wild boars to rural animals was also estimated, suggesting that wild reservoirs could take part in the strain introduction to intensive farms, both directly and through the mediation of rural ones. Although the lack of accurate data on rural-intensive contacts prevents any statistical association, the sharing of employees, fomites and vehicles could be involved in breaking implemented biosecurity measures. The significant mixing of strains collected in different Italian areas might be facilitated by such connections. At the same time, the close relation between Italian and foreign strains, the detection of the same strains in different regions and of diverse strains in the same area confirm the weakness of control measures and the efficacy of viral spreading likely through different pathways.

A reason for concern is the detection, in rural and wild populations, of a PCV-3 strain clade that, although not formally classifiable as a new genotype, diverges from most other PCV-3a strains. Despite the biological features of these strains, as well as their real national and international distribution, are unknown, this finding stresses once more that neglected populations, including rural ones, might host peculiar variants whose behavior and evolution are currently unknown and will require future investigations, representing a potential menace for the intensive production, now or in the future (Molini et al., 2021; Franzo et al., 2021a; Faustini et al., 2023). Although no evidence of a differential action of selective pressures acting on different animal categories was detected in the present study, it must be stressed that the statistical power of the analysis was likely lowered by the limited number of available sequences, especially in light of the low variability of PCV-3.

In fact, the present study is not devoid of limitations, which are largely due to the limited and sparse sequence availability (particularly for rural farms and wild boars) in public datasets. This shortage is magnified by the frequent lack of proper metadata relative to the sampled strains. While the sampling approach was standardized as far as possible in Italy, this could not be guaranteed for other studies and an unavoidable bias due to different diagnostic, sequencing and reporting activities in different countries over time is likely and might have partially affected PCV-3 molecular epidemiology depiction. Other strains or clades with peculiar genetic features could thus have been missed. Therefore, intensive efforts should be encouraged to promote further, more intensive, epidemiological studies based on shared and standardized sampling and analysis protocols among research groups. Since one of the main purposes of the study was to investigate PCV-3 circulation and strain exchange among different animal categories, samples originating from a limited area of Northern Italy only were included in the study due to the remarkable challenges to obtain and collect samples from rural farms and wild animals. Therefore, a better knowledge could be achieved by broadening the involved area, recruiting more farms and planning extensive follow-up.

Overall, the present study reports, both through traditional statistical analysis, molecular epidemiology and phylodynamic reconstruction, an unexpectedly higher PCV-3 circulation in wild boar and rural pig populations compared to commercial ones. A dominant viral flow from these populations to intensive ones was also estimated, supporting their role in the introduction of new strains. Such evidence is boosted by the higher heterogenicity of PCV-3 strain identified in rural and wild animals, including a highly divergent clade that was clearly different from most PCV-3a strains, although not classifiable as a new genotype. Therefore, further investigation should be performed to explore and evaluate the contact points between these populations. An extension of the present approach to other countries would also help to elucidate if the obtained findings represent a peculiarity of the Italian scenario or a general rule.

## Data availability statement

The datasets presented in this study can be found in online repositories. The names of the repository/repositories and accession number(s) can be found in the article/Supplementary material.

## Ethics statement

The study is exempt from ethics approval since all samples from domestic pigs were obtained during routine clinical diagnostic activity at farm or slaughterhouse. Wild boar samples were achieved in the framework of planned culling campaign performed. No sampling or experimental procedure was specifically designed for the study.

## Author contributions

GFr: conceptualization. GFr, GFa, and ML: methodology. GFr and GFa: formal analysis. GFr: resources, writing original draft preparation, and project administration. GB, MDM, and VG: data collection. CT, MLM, MD, and FP: data curation. CT, ML, MLM, and GFr: writing—review and editing. GFr and MC: funding acquisition. All authors have read and agreed to the published version of the manuscript.

## Funding

This work was supported by the Department of Animal Medicine, Production and Health, University of Padua (grant number BIRD225455/22).

## Conflict of interest

The authors declare that the research was conducted in the absence of any commercial or financial relationships that could be construed as a potential conflict of interest.

## Publisher’s note

All claims expressed in this article are solely those of the authors and do not necessarily represent those of their affiliated organizations, or those of the publisher, the editors and the reviewers. Any product that may be evaluated in this article, or claim that may be made by its manufacturer, is not guaranteed or endorsed by the publisher.
