# Supplementary material for: Wilder than intense: higher frequency, variability, and viral flows of porcine circovirus 3 in wild boars and rural farms compared to intensive ones in northern Italy
Source: Front Microbiol. 2023 Jul 31;14:1234393. doi: 10.3389/fmicb.2023.1234393 (PMC10425237; doi:10.3389/fmicb.2023.1234393)
Supplement: Supplementary file 1 [file Data_Sheet_1.PDF]

Supplementary Material

Wilder than intense: Higher frequency, variability and viral flows of Porcine circovirus 3 (PCV-3) in wild boars and rural farms compared to intensive ones in Northern Italy.

Giovanni Franzo[1*], Giulia Faustini[1], Matteo Legnardi[1], Giacomo Berto[2], Mariangela Dal Maso[2], Viviana Genna[3], Maria Luisa Menandro[1], Francesca Poletto[1], Mattia Cecchinato[1], Michele Drigo[1],Claudia Maria Tucciarone[1]

[1] Dept. of Animal Medicine, Production and Health, University of Padova, viale dell'Università 16, Legnaro, PD, 35020, Italy

[2]AULSS 8 Berica, Dip di Prevenzione, Servizi Veterinari, via 4 Novembre, Vicenza

[3] Azienda Ulss 9 Scaligera—Via Valverde, 42-37122 Verona, Italy

*** Correspondence:**
Giovanni Franzo

giovanni.franzo@unipd.it

Supplementary Figure 1. Maximum likelihood phylogenetic tree reconstructed based on the ORF2 (A and B) and complete genome (C and D) of the strains sequenced in the present study plus a set of reference sequences. The animal category (A and C) and geographical location (B and D) have been color-coded. collection host, country and date are reported in the strain name.

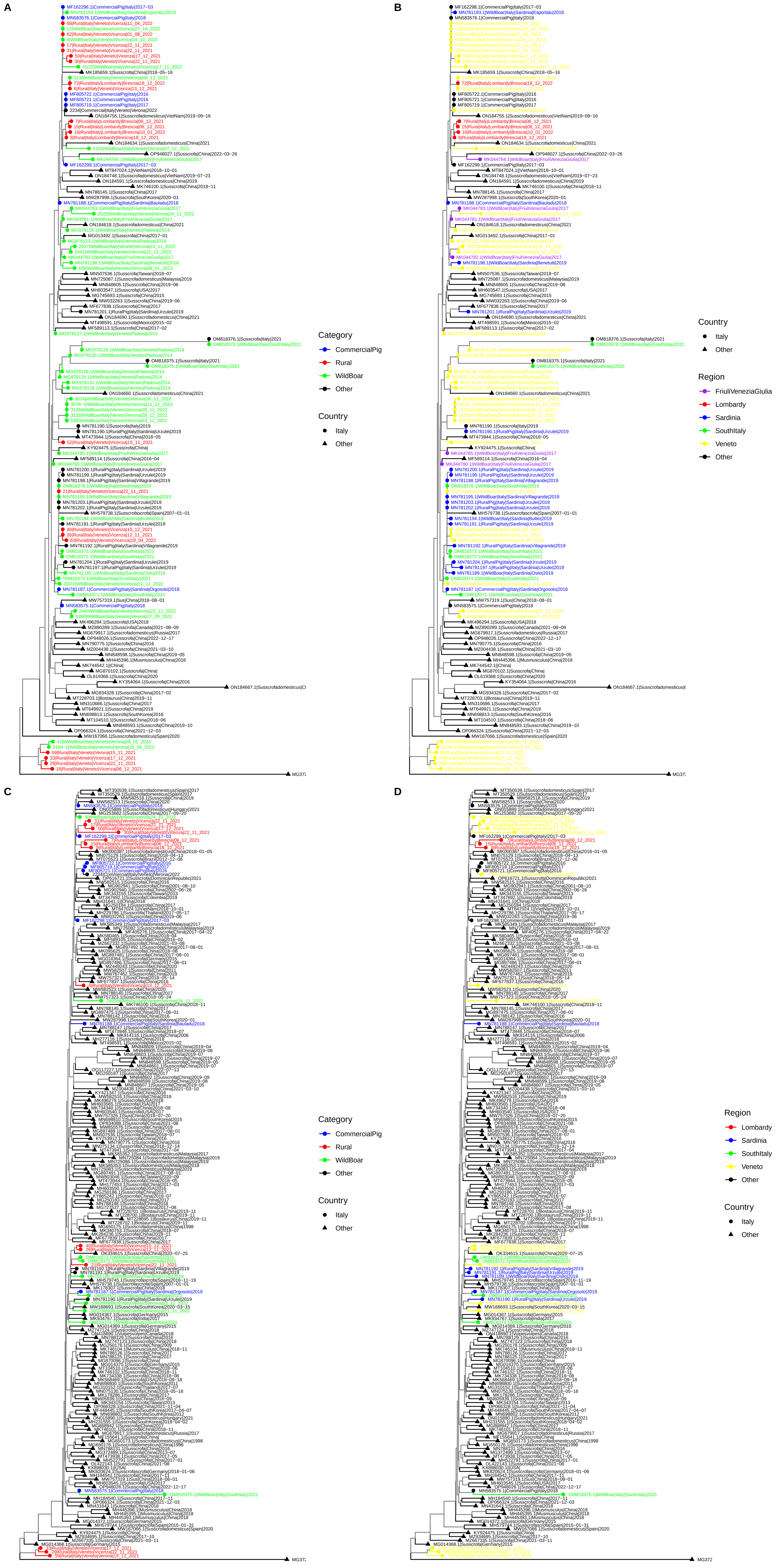
A
B
C
D
Category
CommercialPig
Rural
WildBoar
Other
Country
Italy
Other
Region
FriuliVeneziaGiulia
Lombardy
Sardinia
SouthItaly
Veneto
Other
Category
CommercialPig
Rural
WildBoar
Other
Country
Italy
Other
Region
Lombardy
Sardinia
SouthItaly
Veneto
Other
Country
Italy
Other
