# Supplementary material for: Wilder than intense: higher frequency, variability, and viral flows of porcine circovirus 3 in wild boars and rural farms compared to intensive ones in northern Italy
Source: Front Microbiol. 2023 Jul 31;14:1234393. doi: 10.3389/fmicb.2023.1234393 (PMC10425237; doi:10.3389/fmicb.2023.1234393)
Supplement: Supplementary file 2 [file Data_Sheet_2.PDF]

frontiers

Supplementary Material

Wilder than intense: Higher frequency, variability and viral flows of Porcine circovirus 3 (PCV-3) in wild boars and rural farms compared to intensive ones in Northern Italy.

Giovanni Franzo[1*], Giulia Faustini[1], Matteo Legnardi[1], Giacomo Berto[2], Mariangela Dal Maso[2], Viviana Genna[3], Maria Luisa Menandro[1], Francesca Poletto[1], Mattia Cecchinato[1], Michele Drigo[1],Claudia Maria Tucciarone[1]

[1] Dept. of Animal Medicine, Production and Health, University of Padova, viale dell'Università 16, Legnaro, PD, 35020, Italy

[2]AULSS 8 Berica, Dip di Prevenzione, Servizi Veterinari, via 4 Novembre, Vicenza

[3] Azienda Ulss 9 Scaligera—Via Valverde, 42-37122 Verona, Italy

*** Correspondence:**
Giovanni Franzo

giovanni.franzo@unipd.it

Supplementary table 1. List of sequences obtained in the present study. The accession numbers and the relative metadata are reported.

Acc.Number	Isolate	Host	Breed	Country	Isolation source	Collection_date	Sequence
**OQ754379**	6	Sus scrofa	Rural	Italy	Veneto	13-Dec-2021	Complete Genome
**OQ754380**	8	Sus scrofa	Rural	Italy	Lombardy	18-Dec-2021	Complete Genome
**OQ754381**	17	Sus scrofa	Rural	Italy	Veneto	22-Nov-2021	Complete Genome
**OQ754382**	31	Sus scrofa	Rural	Italy	Veneto	22-Nov-2021	Complete Genome
**OQ754383**	9	Sus scrofa	Wild Boar	Italy	Veneto	24-10-2022	Complete Genome
**OQ754384**	2234	Sus scrofa	Commercial	Italy	Veneto	2022	Complete Genome
**OQ754385**	15	Sus scrofa	Rural	Italy	Lombardy	06-Dec-2021	Complete Genome
**OQ754386**	30	Sus scrofa	Rural	Italy	Veneto	22-Nov-2021	Complete Genome
**OQ754387**	50	Sus scrofa	Rural	Italy	Veneto	17-Dec-2021	Complete Genome
**OQ754388**	21	Sus scrofa	Rural	Italy	Veneto	22-Nov-2021	Complete Genome
**OQ754389**	26	Sus scrofa	Rural	Italy	Veneto	12-Nov-2021	Complete Genome
**OQ754390**	40	Sus scrofa	Rural	Italy	Veneto	10-Dec-2021	Complete Genome
**OQ754391**	20/22	Sus scrofa	Wild Boar	Italy	Veneto	11-11-2022	Complete Genome
**OQ754392**	5363	Sus scrofa	Wild Boar	Italy	Veneto	03-Dec-2022	Complete Genome
**OQ754393**	7	Sus scrofa	Rural	Italy	Lombardy	06-Dec-2021	Complete Genome
**OQ754394**	29	Sus scrofa	Rural	Italy	Veneto	22-Nov-2021	Complete Genome
**OQ754395**	33	Sus scrofa	Rural	Italy	Veneto	17-Dec-2021	Complete Genome
**OQ754396**	59	Sus scrofa	Rural	Italy	Veneto	15-Nov-2021	Complete Genome
**OQ754397**	2925	Sus scrofa	Wild Boar	Italy	Veneto	24-Nov-2022	Complete Genome
**OQ754398**	72	Sus scrofa	Rural	Italy	Lombardy	18-Dec-2022	ORF2
**OQ754399**	313	Sus scrofa	Wild Boar	Italy	Veneto	6-Dec-2022	ORF2
**OQ754400**	16	Sus scrofa	Rural	Italy	Lombardy	10-Jan-2022	ORF2
**OQ754401**	62	Sus scrofa	Rural	Italy	Veneto	1-Aug-2022	ORF2
**OQ754402**	68	Sus scrofa	Rural	Italy	Veneto	11-Apr-2022	ORF2
**OQ754403**	12	Sus scrofa	Wild Boar	Italy	Veneto	27-Oct-2022	ORF2
**OQ754404**	25/22	Sus scrofa	Wild Boar	Italy	Veneto	17-Nov-2022	ORF2
**OQ754405**	420	Sus scrofa	Wild Boar	Italy	Veneto	8-Jan-2023	ORF2
**OQ754406**	2441	Sus scrofa	Wild Boar	Italy	Veneto	22-Dec-2022	ORF2
**OQ754407**	2927	Sus scrofa	Wild Boar	Italy	Veneto	24-Nov-2022	ORF2
**OQ754408**	63	Sus scrofa	Rural	Italy	Veneto	19-Apr-2022	ORF2
**OQ754409**	3133	Sus scrofa	Wild Boar	Italy	Veneto	28-Dec-2022	ORF2
**OQ754410**	3135	Sus scrofa	Wild Boar	Italy	Veneto	28-Dec-2022	ORF2
**OQ754411**	3078_2	Sus scrofa	Wild Boar	Italy	Veneto	11-Jan-2023	ORF2
**OQ754412**	3624	Sus scrofa	Wild Boar	Italy	Veneto	30-Nov-2022	ORF2
**OQ754413**	5390	Sus scrofa	Wild Boar	Italy	Veneto	17-Sep-2022	ORF2
**OQ754414**	2442	Sus scrofa	Wild Boar	Italy	Veneto	22-Dec-2022	ORF2
**OQ754415**	52	Sus scrofa	Rural	Italy	Veneto	15-Nov-2021	ORF2
**OQ754416**	5104	Sus scrofa	Wild Boar	Italy	Veneto	7-Dec-2022	ORF2
**OQ754417**	18	Sus scrofa	Rural	Italy	Veneto	6-Dec-2021	ORF2
**OQ754418**	5109_1	Sus scrofa	Wild Boar	Italy	Veneto	15-Apr-2022	ORF2
**OQ754419**	419	Sus scrofa	Wild Boar	Italy	Veneto	8-Jan-2023	ORF2
